# Indirect evolutionary rescue: prey adapts, predator avoids extinction

**DOI:** 10.1111/eva.12295

**Published:** 2015-08-13

**Authors:** Masato Yamamichi, Brooks E Miner

**Affiliations:** Department of Ecology and Evolutionary Biology, Cornell UniversityIthaca, NY, USA

**Keywords:** climate change, community evolutionary rescue, contemporary evolution, eco-evolutionary dynamics, eco-evolutionary feedback, environmental change, phenotypic plasticity, rapid evolution

## Abstract

Recent studies have increasingly recognized evolutionary rescue (adaptive evolution that prevents extinction following environmental change) as an important process in evolutionary biology and conservation science. Researchers have concentrated on single species living in isolation, but populations in nature exist within communities of interacting species, so evolutionary rescue should also be investigated in a multispecies context. We argue that the persistence or extinction of a focal species can be determined solely by evolutionary change in an interacting species. We demonstrate that prey adaptive evolution can prevent predator extinction in two-species predator–prey models, and we derive the conditions under which this indirect evolutionary interaction is essential to prevent extinction following environmental change. A nonevolving predator can be rescued from extinction by adaptive evolution of its prey due to a trade-off for the prey between defense against predation and population growth rate. As prey typically have larger populations and shorter generations than their predators, prey evolution can be rapid and have profound effects on predator population dynamics. We suggest that this process, which we term ‘indirect evolutionary rescue’, has the potential to be critically important to the ecological and evolutionary responses of populations and communities to dramatic environmental change.

## Introduction

The interaction between ecological and evolutionary processes is now recognized as having fundamental importance in numerous natural communities and will likely become increasingly relevant as the pace of global change increases (Ellner 2013; Carlson et al. 2014). The concept of evolutionary rescue identifies situations in which a population avoids extinction following adverse environmental change by rapidly adapting to its altered environment (Gomulkiewicz and Holt 1995; Kinnison and Hairston 2007; Gonzalez et al. 2013; Alexander et al. 2014; Carlson et al. 2014). This is possible when the positive effect of adaptive evolution on population rate of change is greater than the negative effect of the altered environment.

Since its genesis, a primary motivation behind the study of evolutionary rescue has been its relevance and potential utility in conservation applications (Kinnison and Hairston 2007). This research encompasses the more specific concepts of genetic rescue, which is limited to situations in which beneficial alleles that help rescue a population from extinction are introduced via immigration (Whiteley et al. 2015), and assisted gene flow, a still narrower definition in which immigrants are actively introduced by managers (Aitken and Whitlock 2013). Common to all studies of evolutionary rescue is a focus on small, imperiled populations of an individual species typically threatened by rapid environmental change. Existing studies have largely considered a single species evolving in response to an abiotic challenge (reviewed in Alexander et al. 2014; Carlson et al. 2014) and have examined how the probability of rescue is affected by the rate of environmental change (Lindsey et al. 2013), initial population size (Bell and Gonzalez 2009), founding genetic variation (Agashe et al. 2011), spatial structure (Bell and Gonzalez 2011), phenotypic plasticity (Chevin et al. 2010), and genetic architecture underlying adaptation (Orr and Unckless 2008). If the study of evolutionary rescue is to have meaningful conservation applications, it must be also investigated in a multispecies context, because outside the laboratory all species exist within communities of interacting species. Fortunately, this line of investigation has recently been pioneered using mathematical models (Jones 2008; Norberg et al. 2012; Fussmann and Gonzalez 2013; Kovach-Orr and Fussmann 2013; Northfield and Ives 2013; Osmond and de Mazancourt 2013).

We propose a new mechanism of evolutionary rescue in the community context: that a *non*evolving predator can be rescued from extinction *solely due to the evolution of its prey*. This overlooked and seemingly counterintuitive outcome, which we term ‘indirect evolutionary rescue’, has a logical mechanistic basis with empirical evidence, and should be explored in future studies of community responses to environmental change. The mechanism occurs when there is a fitness cost to prey of defense against predation, such that defense declines when predators are scarce. An environmental perturbation that increases predator mortality then leads to reduced predator population size, which selects for prey with reduced defense; this indirectly increases the population growth rate of predators feeding on those prey. This set of interactions can rescue a predator population from extinction whenever the benefit to predator growth rate due to reduced prey defense is greater than the negative effect on the predators of the environmental perturbation. Adaptive phenotypic plasticity of defense traits (i.e., inducible defenses) can have a qualitatively similar effect on predator persistence as adaptive defense evolution (‘indirect plastic rescue’), although the faster response of inducible defense to environmental change may result in quantitative differences (Yamamichi et al. 2011; Kovach-Orr and Fussmann 2013).

The idea that prey defense adaptation will affect predator population dynamics is not without precedent. Theoretical studies in the context of fisheries management found that evolution of prey defense can increase predator population size even as predator mortality increases (i.e., ‘the Hydra effect’; Abrams and Matsuda 2005; Schröder et al. 2014). Yet the Hydra effect and indirect evolutionary rescue are different concepts, as the former indicates that increasing predator mortality can increase predator abundance, whereas indirect evolutionary rescue occurs when prey evolution prevents predator extinction. Although Abrams (2009) briefly mentioned situations in which prey adaptation could increase the maximum mortality at which predators could persist, our goal in this study is to obtain the general mathematical condition for indirect rescue to occur and to draw attention to the indirect rescue phenomenon as relevant in the broader context of community eco-evolutionary responses to environmental change. Furthermore, we propose that indirect evolutionary rescue can occur not only with adaptive defenses, but also during competitive interactions and adaptive foraging (Appendices S1 and S2).

Below we present a mathematical model illustrating indirect evolutionary rescue and identify a previously unappreciated role of this mechanism in a recent theoretical study of coevolution's role in the persistence of interacting species (Northfield and Ives 2013). Finally, we review existing empirical evidence supporting the occurrence and importance of indirect evolutionary rescue.

## Model

To illustrate our conceptual framework, we consider a general predator–prey model with prey evolution. The model has logistic growth of prey (*N*), the Holling type I (linear) functional response of predator (*P*), and adaptive evolution of a prey trait (*x*) defined using a quantitative trait model,


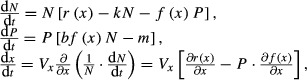
(1)

where the prey quantitative trait, *x*, determines the predator attack rate on prey, *f*(*x*), and the intrinsic rate of increase of the prey population, *r*(*x*). There is a trade-off between growth (*r*) and defense against predation (*f*): larger *x* decreases successful predation, but simultaneously decreases prey growth (*f*(*x*) and *r*(*x*) are decreasing functions of *x*: 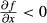 and 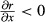). The quantitative trait model assumes that the trait value of the prey population changes whenever it increases fitness (the per capita population growth rate) as a function of population size and trait value along fitness gradients with constant additive genetic variance, *V*_*x*_ (Lande 1976; Abrams 2001). Density-dependent prey growth depends on the parameter *k*, *b* is the predator conversion efficiency, and *m* is the predator mortality rate.

### Coevolution model

Although we demonstrate that indirect evolutionary rescue is possible using the above equation, a more interesting and ecologically relevant question concerns how important it is relative to evolutionary rescue as typically defined, which we here term ‘direct evolutionary rescue’ for clarity (Table 1). The relative importance of indirect evolutionary rescue can be exemplified using a modified predator–prey model with coevolution (Tien and Ellner 2012). The model is described as

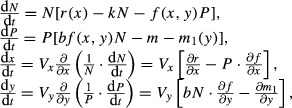
(2)

where the predator now has a quantitative trait *y*, which modifies both attack rate on prey and predator mortality. There is a trade-off for the predator between prey consumption and mortality: increasing the value of trait *y* increases the attack rate, *f*(*x*, *y*) (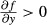), and simultaneously leads to higher predator mortality (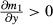). Thus, predator and prey each exhibit trait trade-offs: increasing prey defense *x* decreases predation rate, but comes with a growth cost to the prey. Increasing predator counter-defense *y* increases predation rate, but comes with a mortality cost to the predator. *V*_*x*_ and *V*_*y*_ are the additive genetic variances for prey and predator traits, respectively.

**Table 1:** The fate of a predator population in face of abrupt environmental change

	No predator evolution	Predator evolution
No prey evolution	Extinction	Direct evolutionary rescue
Prey evolution	Indirect evolutionary rescue	Coevolutionary rescue

## Results

**Figure 1:**
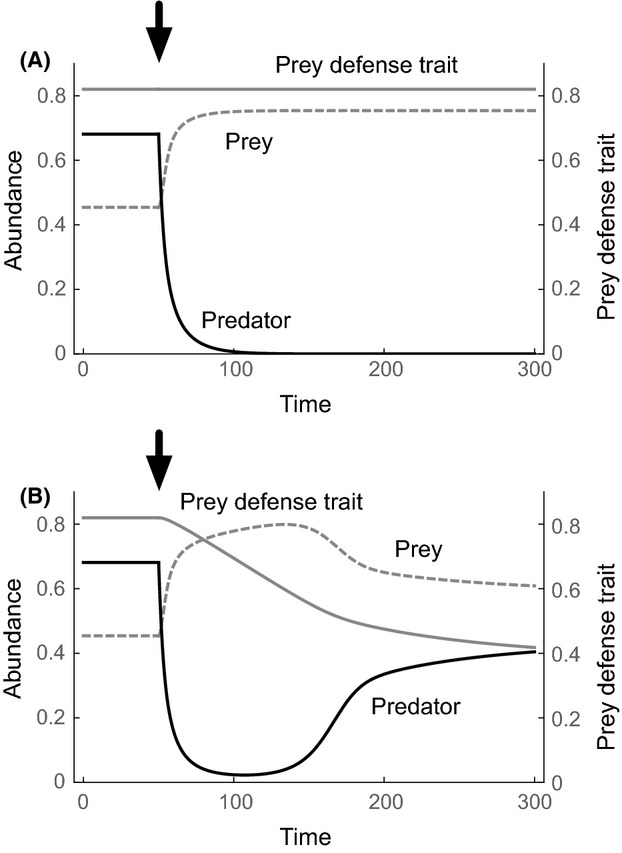
Indirect evolutionary rescue in eqn (1). An abrupt environmental change occurs when *t* = 50 as indicated by arrows (the predator mortality, *m*, changes from 0.2 to 0.4). Without prey evolution, the predator goes extinct (A), whereas when prey can evolve, the predator population increases after its initial decline (B). Adaptive evolution lowers prey defense (B), which stays constant in the case of no evolution (A). Black solid lines: predator abundance, gray dashed lines: prey abundance, and gray solid lines: prey defense trait. Parameter values are *a* = 0.3, *G* = *k* = *b* = 1, and *V*_*x*_ = 0 (A) or 0.01 (B). The predator and prey abundances and the prey trait reached an equilibrium before the environmental change with *V*_*x*_ > 0.

We demonstrate indirect evolutionary rescue using numerical simulations with eqn (1) assuming that *f*(*x*) = *G*e^−*x*^ and *r*(*x*) = 1 – *ax*, where *G* is the attack rate coefficient and *a* is the defense cost coefficient. We chose a linear function for the intrinsic rate of increase as it can be negative or positive, and an exponential function for the attack rate because it should be always positive. The mechanism underlying indirect evolutionary rescue is as follows: consider a situation in which a predator and its prey experience environmental change that is detrimental to the predator (in this simulation, increased predator mortality) and results in its extinction in the absence of evolutionary change (Fig. 1A). When the prey species exhibits a trade-off between defense against predation and maximum population growth rate (i.e., intrinsic rate of increase), environmental change that is detrimental to the predator results in reduced predation pressure on the prey due to decreased predator abundance (Fig. 1B). Because of its defense/growth rate trade-off, the prey then evolves toward a less defended phenotype with a higher intrinsic rate of increase (Fig. 1B). The reduction in prey defense consequently permits the persistence of the predator, even though environmental conditions are not favorable to the predator, and the predator population itself has not evolved (Fig. 1B). Although at first counterintuitive, the result of this interaction is that adaptive evolution by a prey species to increase its population growth rate causes the persistence of its predator.

We show the general mathematical condition for indirect rescue to occur without assuming specific functions for predation (*f*) and growth (*r*). As abrupt environmental change increases predator mortality and eventually causes its extinction, an important value for evaluating evolutionary rescue is the maximum value of predator mortality *m* at which predator can persist (that is, its abundance is nonzero). We call this 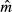, which equals *rbf*/*k*, because at a stable equilibrium, predator and prey abundances are 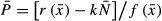 and 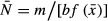, respectively. Increasing predator mortality *m* decreases 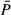 and eventually causes predator extinction when 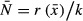. The mortality 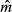 is a decreasing function of the prey trait *x*, because *bf*/*k* and *r* are both decreasing functions of *x* due to the trade-off between defense and growth. The prey population evolves to reduce defense (*x* decreases) when *m* increases, because at a stable equilibrium, the right-hand side of d*x*/d*t* in eqn (1) becomes negative with decreased *P*. Prey evolution therefore increases 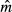, the maximum predator mortality rate at which the predator population is viable; this increase in 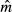 is the quantitative contribution of indirect evolutionary rescue. This result also holds under the Holling type II (saturating) functional response for the predator.

### Coevolution model

In the coevolution model, the maximum value of predator mortality *m* at which predator abundance is nonzero now includes *m*_1_: 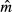 = *rbf*/*k* – *m*_1_. Therefore, the relationship between 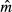, the maximum mortality at which the predator can persist, and *y*, the predator's counter-adaptation to prey defense, is:


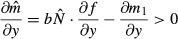
(3)

where 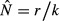, and 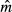 is an increasing function of *y* (because 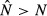 and the right-hand side of d*y*/d*t* in eqn (2) is zero at the coexistence equilibrium). The predator population evolves to increase counter-defense (*y* increases) when *m* increases, because at a stable equilibrium, the right-hand side of d*y*/d*t* in eqn (2) becomes positive with increased *N*. Therefore, predator evolution can prevent its extinction (direct evolutionary rescue is possible). Below we present an example in which indirect evolutionary rescue is more important than direct rescue even when both predator and prey traits evolve.

**Figure 2:**
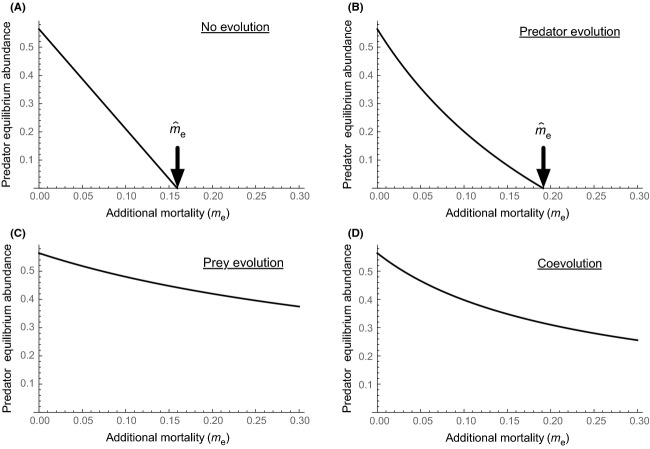
Effects of predator evolution, prey evolution, or both on predator abundance as a function of the magnitude of environmentally imposed predator mortality (*m*_e_) in eqn (2). *X*-axis is additional predator mortality due to environmental change (*m*_e_), and *Y*-axis is predator equilibrium abundance. Black arrows represent the maximum environmentally imposed mortality at which the predator can persist (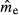). Parameter values are *m*_0_ = 0.2, *c* = 2, and *V*_*x*_ = *V*_*y*_ = 0 or >0, with all other parameters the same as in Fig. 1. (A): No evolution (*V*_*x*_ = *V*_*y*_ = 0). (B): Predator evolution only (*V*_*x*_ = 0 and *V*_*y*_ > 0). (C): Prey evolution only (*V*_*x*_ > 0 and *V*_*y*_ = 0). (D) With both predator and prey evolution (*V*_*x*_ > 0 and *V*_*y*_ > 0).

As in the previous model, *r*(*x*) is a decreasing function of *x,* and here we assume *f*(*x*, *y*) = *G*e^(*y* – *x*)^, *r*(*x*) = 1 – *ax*, and *m*_1_(*y*) = e^*cy*^ for the following analyses, where *G* is the attack rate coefficient, *a* is the prey defense cost coefficient, and *c* is the predator counter-defense cost coefficient. We chose an exponential function for the predator cost function because it should be always positive. We assume that predator mortality *m* consists of a basal mortality *m*_0_ under reference environmental conditions, combined with an additional mortality *m*_e_ due to abrupt environmental change that is detrimental to the predator (thus, *m* = *m*_0_ + *m*_e_). We explore the effects of predator evolution, prey evolution, or both on predator abundance following increased predator mortality due to sudden environmental change (Fig. 2). We first calculate equilibrium abundances and trait values when both traits can evolve and *m*_0_ = 0.2 and *m*_e_ = 0, and then apply additional mortality with a range of positive values for *m*_e_ to evaluate the relative importance of indirect versus direct evolutionary rescue.

Without evolution in either predator or prey, the predator goes extinct when additional mortality imposed by environmental change exceeds 0.16 (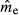
≈ 0.16; Fig. 2A); adding predator evolution slightly increases the maximum mortality at which the predator can persist, due to an increase in *y* (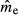 ≈ 0.19: Fig. 2B). In contrast, prey evolution leads to an increase in the maximum mortality at which predators can persist, because the prey population decreases defense (by decreasing *x* value) to increase its intrinsic rate of increase: the predator does not go extinct when *m*_e_ < 0.3 (Fig. 2C). Evolution of both predator and prey together also prevents extinction when *m*_e_ < 0.3 (Fig. 2D). This demonstrates that it is possible for prey evolution (Fig. 2C) to be more important than predator evolution itself (Fig. 2B) in the framework of predator–prey coevolution models (Tien and Ellner 2012), which is the central message of ‘indirect evolutionary rescue’. This finding should not discount the role of direct evolutionary rescue, and we note that the relative importance of indirect versus direct rescue depends on the trade-off associated with the predator's increase in growth rate. Parameter values certainly affect these outcomes; for example, decreasing *c* (cost of predator counter-defense) results in stronger influence of predator evolution on 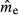. However, indirect evolutionary rescue is a general phenomenon as long as the prey exhibits a trade-off between population growth rate and defense, which has been observed in various species, as we describe below. In addition to adaptation in prey defense, we also provide theoretical examples of indirect evolutionary rescue via adaptation in predator foraging (Matsuda et al. 1996; Kondoh 2003) and in competition mediated by chemical allelopathy (Mougi 2013) (Appendices S1 and S2).

### Comparison to previous studies

The role of evolutionary responses to environmental change within a community context is highlighted by a recent theoretical study, which suggests that predator–prey coevolution can prevent predator extinction following environmental change in a discrete-time predator–prey model with evolving quantitative traits (Northfield and Ives 2013). These authors examined models in which environmental change affected either prey growth rate or predation rate, and in both cases, they concluded that coevolution prevented predator extinction following detrimental environmental change. Notably, however, these authors did not explore situations where only one of the two interacting species can evolve, and their findings do not indicate whether prey or predator evolution alone (rather than coevolution) is sufficient to rescue the predator from extinction.

**Figure 3:**
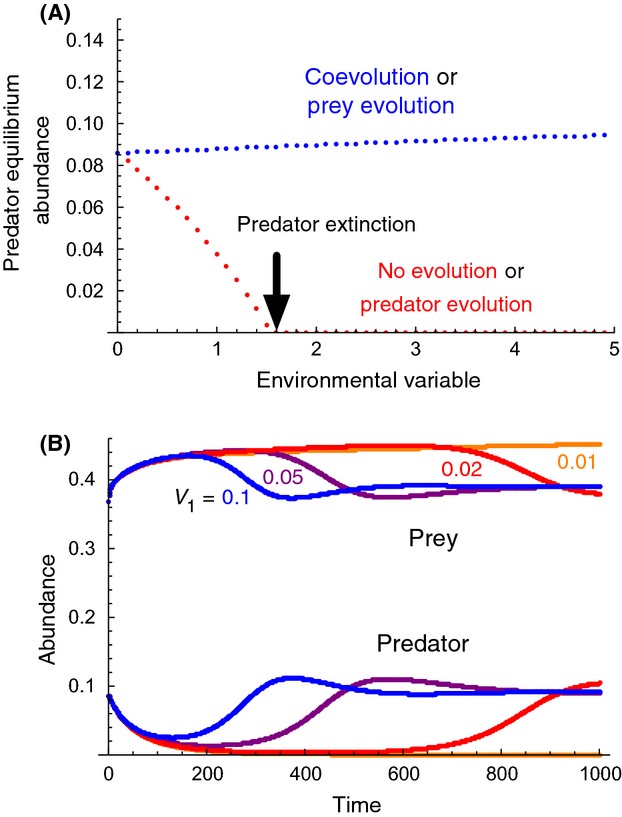
Effects of predator evolution, prey evolution, or both, on abundances following an environmental change that negatively affects predation rate. (A): *X*-axis is the environmental variable; positive values of larger magnitude cause larger decreases in predation rate. *Y*-axis is predator equilibrium abundance. Additive genetic variance of evolving traits in prey (*V*_1_) and predator (*V*_2_) is either 0 (no evolution) or 1 (with evolution). Red dots: no evolution (*V*_1_ = *V*_2_ = 0), or with predator evolution only (*V*_1_ = 0, *V*_2_ = 1), blue dots: with prey evolution only (*V*_1_ = 1, *V*_2_ = 0), or with both predator and prey evolution (*V*_1_ = *V*_2_ = 1). Note that the environmental variable was multiplied by −1 to be consistent with Fig. 2. Other parameters match those of Fig. 4 of Northfield and Ives (2013). (B): Effects of prey additive genetic variance on rescue of the predator following an environmental change detrimental to the predator (an abrupt change from 0 to 3 on the *X*-axis of 3A). Additive genetic variance of prey (*V*_1_) is 0.1 (blue), 0.05 (purple), 0.02 (red), or 0.01 (orange), whereas that of predator (*V*_2_) is 0.

To evaluate the relative roles of indirect and direct rescue within the model framework of Northfield and Ives (2013), we examined cases in which only the prey, or only the predator, is permitted to evolve. We found that the occurrence of rescue depended on indirect effects: prey evolution alone is sufficient to rescue the predator from extinction, whereas predator evolution alone cannot prevent extinction (Fig. 3) using the same parameter values as the original study. This outcome was consistent under scenarios where environmental change affected prey growth rate (data not shown) or predation rate (Fig. 3A). This is not direct evolutionary rescue; rather, it is indirect evolutionary rescue because extinction of the predator is prevented by prey evolution, not by predator evolution. We therefore suggest a subtle yet important modification of the conclusions of Northfield and Ives (2013) with respect to predator–prey interactions: the fundamentally important process in their model is not coevolution *per se*; rather, the indirect effect of prey evolution is the cause of predator persistence in the face of detrimental environmental change.

For a specific case of the model of Northfield and Ives (2013), we examined the effects of prey additive genetic variance on the minimum population size experienced by the predator population at the bottom of the U-shaped trajectory of evolutionary rescue (Fig. 3B). We found that higher prey genetic variance, and the increasingly rapid prey evolution that results from it, shortens the predator population's vulnerable period of extremely low abundance, when extinction would be likely due to demographic stochasticity.

## Discussion

We apply the label ‘indirect evolutionary rescue’ because adaptive evolution of an interacting species (in our example, the prey) rescues a focal species (the predator) from extinction. Additional analyses suggest that indirect evolutionary rescue can occur not only with adaptive defense, but also in other interspecific interactions with conflicting interests including competitive interactions and adaptive foraging (Appendices S1 and S2). Indirect evolutionary rescue can be regarded as an interspecific indirect genetic effect (IIGE; Shuster et al. 2006), in which the genetic composition of one species affects an interacting species. Such effects have been intensively studied in the context of herbivore–plant interactions (community genetics; Bailey et al. 2009), but have not yet been recognized in the context of evolutionary rescue.

Indirect evolutionary rescue in predator–prey systems is generally possible whenever prey phenotypes exhibit a trade-off between defense against predation and population growth rate, as long as genetic variance for these traits is present. We have analyzed three models to demonstrate our hypothesis, but the principle at work is general: we predict similar dynamics whenever a cost of defense for prey means that reduced predator abundance will lead to reductions in prey defense. Empirical trade-offs between growth and defense exist for algae (Yoshida et al. 2004; Becks et al. 2010; Kasada et al. 2014), land plants (Koricheva 2002; Fine et al. 2006), and microbes (Gagneux et al. 2006; Andersson and Hughes 2010). Microcosm experiments revealed that decreases in predator abundance were followed by prey rapidly abandoning defenses, via either evolution (Becks et al. 2010) or phenotypic plasticity (Verschoor et al. 2004). Indeed, such trade-offs are the basis for the evolution of inducible defense (Tollrian and Harvell 1999).

Invasive species have been hypothesized to show rapid evolution toward lower defense and faster growth when they invade novel, enemy-free environments, as in the EICA (Evolution of Increased Competitive Ability) hypothesis (Blossey and Nötzold 1995; Leishman et al. 2014). On the other hand, reintroduction of predators can increase prey defense traits along with a concomitant cost of reduced reproduction, as shown in the wolf-elk system in Yellowstone (Creel et al. 2007). A trade-off between defense and population growth is also possible with regard to allocation of time: for example, freshwater copepods can avoid seasonal fish predation by producing diapausing eggs before fish become active, but to do so they must sacrifice their current reproductive output of nondiapausing (immediately hatching) eggs (Hairston and Munns 1984; Ellner 2013). Note that the specific shape of the trade-off relationship can also change the effectiveness of rescue: indirect evolutionary rescue is more likely when prey employ a specific defense against a single predator species. In contrast, prey coexisting with multiple predators may show general defenses against all enemies, which would weaken the effect of indirect evolutionary rescue.

Shifts in the abundances of multiple prey species affect a predator population in the same manner as quantitative trait variation of a single prey species. Therefore, in addition to genetic variation within prey species, prey species diversity (Abrams and Matsuda 2005; Abrams 2009) and phenotypic plasticity (Yamamichi et al. 2011; Kovach-Orr and Fussmann 2013) are surely important for predator persistence in the face of detrimental environmental change. Indeed, the quantitative trait models we use (eqns (1) and (2)) have been used by others to represent phenotypic plasticity and genetic evolution by changing the additive genetic variance parameter (Abrams et al. 1993; Taylor and Day 1997; although there are other ways to represent inducible defense: Ramos-Jiliberto 2003; Vos et al. 2004). Therefore, inducible defense and adaptive defense evolution would have the similar effects on predator persistence (as direct evolutionary rescue and direct plastic rescue: Chevin et al. 2010), but the faster response of inducible defense to environmental change may slow down the initial population decline and result in the shallower U-shaped demographic trajectory and larger minimum density comparing to those of evolutionary rescue (Kovach-Orr and Fussmann 2013).

The results of a recent empirical study by Kasada et al. (2014) suggest the occurrence indirect evolutionary rescue. For their rotifer–algae microcosm system, a parameterized model predicts that a defended prey genotype causes predator extinction, whereas the presence of prey genetic variation for a trade-off between defense and competitive ability results in predator persistence (with extinction of the defended prey genotype). Experiments by Kasada et al. (2014) verify the latter prediction, but the authors did not conduct an experiment to confirm the former prediction. Compelling empirical evidence of indirect evolutionary rescue could be obtained by manipulating genetic variation of prey populations and observing its effect on predator persistence.

Particularly in the context of conservation applications, it will be important to investigate the relative contributions of both prey evolution and predator evolution to the rescue of imperiled populations from extinction. In predator–prey systems, prey typically exhibit larger population sizes and shorter generation times than their predators, increasing the probability of adaptive evolution by prey populations (Hiltunen et al. 2014). This reality increases the relative importance of indirect evolutionary rescue. On the other hand, in host–parasite and plant–herbivore systems direct evolutionary rescue may be more influential because of the small population sizes and long generation times of the victims (although phenotypic plasticity of victims may play an important role in rescue; Kovach-Orr and Fussmann 2013). We therefore suggest that indirect evolutionary rescue may be more important to the conservation of threatened vertebrate populations, whereas direct evolutionary rescue is likely a more important mechanism for epidemiological and agricultural systems relating to bacteria and insect populations evolving in response to antibiotic or pesticide exposure.

Conservation science typically focuses on the abundance and genetic diversity of focal threatened populations, but indirect evolutionary rescue highlights the importance of biotic interactions to population persistence. Our demonstration that rescue from extinction can be enabled by evolution in an interacting species suggests that the genetic diversity of other, nonthreatened species could be relevant to the persistence of an imperiled species. We predict that such a situation would be most likely to arise when interactions are tightly coupled, such as a consumer that relies on a single resource species rather than having a diverse diet. Because the contribution of indirect evolutionary rescue depends on the presence of genetic variation in an interactor species, low abundance of the interactor could limit indirect rescue. Situations in which both a focal species and its interactor have declined should be more worrisome than those in which the interactor is still abundant. Conversely, when the interactor population is large, indirect rescue is more likely and should not be overlooked. The probability of indirect rescue may also be higher when interactor populations receive regular immigrants from a genetically distinct population; this is extends the concept of genetic rescue (Whiteley et al. 2015) discussed earlier. Finally, we can also extend the concept of assisted gene flow (Aitken and Whitlock 2013): active management or introduction of a strongly interacting species may assist conservation of a threatened population due to indirect rescue.

Indirect evolutionary rescue is a concept that only becomes apparent when community ecology and evolutionary biology are merged, and it has potential applications in yet a third discipline, conservation science. The phenomenon has not been thoroughly investigated theoretically and has yet to be directly addressed in an empirical study system. We encourage further study of this intriguing mechanism of population persistence in the face of environmental change.

## Supporting Information

**Appendix S1.** An example of indirect evolutionary rescue in competitive interactions. **Appendix S2.** An example of indirect evolutionary rescue by adaptive foraging. **Figure S1.** An example of indirect evolutionary rescue in competitive interactions. **Figure S2.** Bifurcation plots of indirect evolutionary rescue in competitive interactions. **Figure S3.** An example of indirect evolutionary rescue by adaptive foraging. **Figure S4.** Bifurcation plots of indirect evolutionary rescue by adaptive foraging.
