# Supplementary material for: Indirect evolutionary rescue: prey adapts, predator avoids extinction
Source: Evol Appl. 2015 Aug 13;8(8):787–95. doi: 10.1111/eva.12295 (PMC4561568; doi:10.1111/eva.12295)
Supplement: Supplementary file 1 [file eva0008-0787-sd1.pdf]

APPENDIX S1. AN EXAMPLE OF INDIRECT EVOLUTIONARY RESCUE IN COMPETITIVE INTERACTIONS

We assume a Lotka-Volterra competition model with allelopathic adaptation after Mougi (2013),

$$\frac{dN_i}{dt} = N_i\left[r_i(u_i) - N_i - \alpha_{ij}(u_i - u_j)N_j\right], \qquad (i = 1, 2) \qquad \text{(S1)}$$
$$\frac{du_i}{dt} = V_i \frac{\partial}{\partial u_i}\left(\frac{1}{N_i}\cdot\frac{dN_i}{dt}\right),$$

where N_i is population density of species i, r_i is the intrinsic rate of increase, and α_{ij} is the competition coefficient. In the chemical allelopathy scenario, the coefficient α_{ij} is a decreasing function of the difference between the traits u_i and u_j ($\frac{\partial \alpha_{ij}}{\partial u_i} < 0$ and $\frac{\partial \alpha_{ij}}{\partial u_j} > 0$). This implies that greater differences in trait values result in larger negative effects on the other species. The cost of allelopathy is incorporated by assuming that r_i is a decreasing function of u_i ($\frac{\partial r_i}{\partial u_i} < 0$). For adaptive evolution of u_i, quantitative trait evolution model along the fitness gradient with the additive genetic variance, V_i, is used (Abrams 2001). The evolutionary dynamics of the trait u_i is then described by

$$\frac{du_i}{dt} = V_i\left(\frac{\partial r_i}{\partial u_i} - N_j\frac{\partial \alpha_{ij}}{\partial u_i}\right). \qquad \text{(S2)}$$

The equilibrium density of species i is $\bar{N}_i = \frac{r_i - r_j\alpha_{ij}}{1 - \alpha_{ij}\alpha_{ji}}$. When the intrinsic rate of increase is represented by $r_i = r_{0i}f(u_i)$, where r_{0i} is the basal intrinsic rate of increase, decreasing r_{0i} by an abrupt environmental change causes extinction of species i when $r_{0i} = r_j\alpha_{ij}/f(u_i)$, which is an extinction threshold that we label as r_{0i}^*. We examine whether evolution of the trait of interacting species, u_j, can prevent extinction (i.e., it can decrease the extinction threshold, r_{0i}^*). Decreasing r_{0i} (and consequently N_i) causes u_j to decrease from equation S2. Therefore, if the extinction threshold is an increasing function of u_j, evolution of the interacting species can prevent extinction. Hence

$$
\begin{aligned}
\frac{\partial r_{0i}^*}{\partial u_j} &= \frac{1}{f(u_i)} \cdot \frac{\partial (r_j \alpha_{ij})}{\partial u_j} \\
&= \frac{1}{f(u_i)} \left(\alpha_{ij} \frac{\partial r_j}{\partial u_j} + r_j \frac{\partial \alpha_{ij}}{\partial u_j} \right),
\end{aligned} \tag{S3}
$$

and indirect evolutionary rescue is possible when $\frac{1}{f(u_i)} \left(\alpha_{ij} \frac{\partial r_j}{\partial u_j} + r_j \frac{\partial \alpha_{ij}}{\partial u_j} \right) > 0$.

We show an example of indirect evolutionary rescue in competitive interactions in numerical simulations. In the following simulations, we assume a function $\alpha_{ij} = \alpha_{0i} \exp(u_i - u_j)$ for the competition coefficient so that it is always positive, where α_{0i} is the basal competition coefficient. The intrinsic rate of increase is represented by a linear function, $r_i = r_{0i}(1 - au_i)$, where r_{0i} is the basal intrinsic rate of increase and a is the defense cost coefficient. We assume that $r_2 = r_{20} - r_{2e}$, where r_{20} is the basic intrinsic rate of increase and r_{2e} is reduction in the intrinsic rate of increase due to an abrupt environmental change. When the basal intrinsic rate of increase of species 2 (r_{02}) changed from 1 to 0.2 ($r_{2e} = 0.8$), species 2 goes extinct without evolution (Fig. S1A). Evolution of the trait of species 2 does not affect the outcome (Fig. S1B), but evolution of the trait of species 1 rescues species 2 from extinction (indirect evolutionary rescue). Coevolution of two species can also avoid extinction of species 2 (Fig. S1D). The bifurcation plots along r_{2e} indicate that adaptive chemical allelopathy of interacting species (species 1) can increase the maximum reduction in the intrinsic rate of increase at which species 2 can persist (Fig. S2).

APPENDIX S2. AN EXAMPLE OF INDIRECT EVOLUTIONARY RESCUE BY ADAPTIVE FORAGING

We assume a diamond food web model (one-predator-two-prey-one-resource) model with adaptive foraging after Matsuda et al. (1996) and Kondoh (2003),

$$\begin{aligned} \frac{dN_i}{dt} &= N_i\left(r_i - N_i - \alpha_{ij}N_j - e_iG_iP\right), \\ \frac{dP}{dt} &= P\left(\sum_{i=1}^{2} b_ie_iG_iN_i - m\right), \qquad (i = 1, 2) \qquad \text{(S4)} \\ \frac{de_i}{dt} &= V_ie_i\left(b_iG_iN_i - \sum_{j=1}^{2} e_jb_jG_jN_j\right), \end{aligned}$$

where N_i is population density of prey species i, P is predator population density, r_i is the intrinsic rate of increase, α_{ij} is the competition coefficient, G_i is the maximum attack rate on prey i, b_i is the conversion efficiency, and m is the predator mortality. The predator foraging effort allocated to prey i is e_i, and is adaptively changing according to prey densities. Because there are only two prey species and $\sum_{i=1}^{2} e_i = 1$, the adaptive dynamics of the foraging trait e_1 is described by

$$\begin{aligned} \frac{de_1}{dt} &= V_1e_1\left(1-e_1\right)\frac{\partial}{\partial e_1}\left(\frac{1}{P}\cdot\frac{dP}{dt}\right) \\ &= V_1e_1\left(1-e_1\right)\left(b_1G_1N_1 - b_2G_2N_2\right). \end{aligned} \qquad \text{(S5)}$$

We show an example of indirect evolutionary rescue by adaptive foraging in the following analyses. We assume that $r_2 = r_{20} - r_{2e}$, where r_{20} is the basic intrinsic rate of increase and r_{2e} is reduction in the intrinsic rate of increase due to an abrupt environmental change. In the following simulations, we assume $b_1 = b_2 = d = 1$, $r_1 = G_1 = 2$, $r_{20} = G_2 = 1.5$; therefore, species 1 is undefended prey and species 2 is defended prey. When the intrinsic rate of increase of species 2 (r_2) changes from 1.5 to 1 ($r_{2e} = 0.5$), this species goes extinct without evolution (Fig. S3A). Adaptive foraging of the predator rescues species 2 from extinction (indirect evolutionary rescue) and this occurs because of reduced predation effort on species 2 (Fig. S3B). The bifurcation plots along r_{2e} indicate that adaptive foraging can increase the maximum reduction in the intrinsic rate of increase at which defended prey can persist (Fig. S4).

LITERATURE CITED

Abrams, P. A. 2001. Modelling the adaptive dynamics of traits involved in inter- and intraspecific interactions: An assessment of three methods. Ecology Letters 4: 166-175.

Kondoh, M. 2003. Foraging adaptation and the relationship between food-web complexity and stability. Science 299: 1388-1391.

Matsuda, H., M. Hori, and P. A. Abrams. 1996. Effects of predator-specific defence on biodiversity and community complexity in two-trophic-level communities. Evolutionary Ecology 10: 13-28.

Mougi, A. 2013. Allelopathic adaptation can cause competitive coexistence. Theoretical Ecology 6: 165-171.

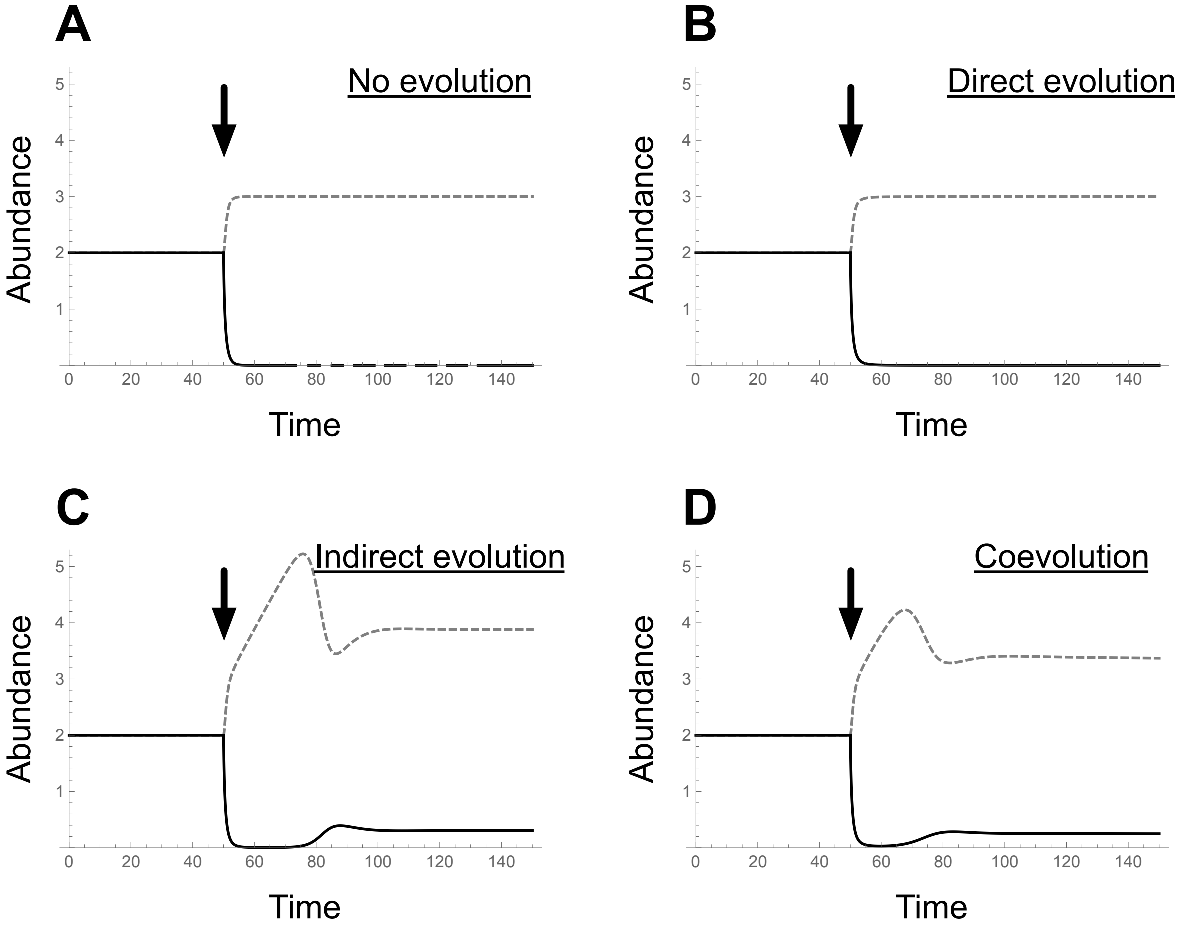

Figure S1. An example of indirect evolutionary rescue in competitive interactions. The basal intrinsic rate of increase of species 2 (r_{02}) changed from 1 to 0.2 at the timing of arrows. Gray dashed lines: species 1 abundance, black solid lines: species 2 abundance. (A) No evolution (V_1 = 0 and V_2 = 0). (B) Direct evolution (V_1 = 0 and V_2 = 0.1). (C) Indirect evolution (V_1 = 0.1 and V_2 = 0). (D) Coevolution (V_1 = 0.1 and V_2 = 0.1). Other parameter values are r_{01} = a = 1 and $\alpha_{01} = \alpha_{02} = 0.5$.

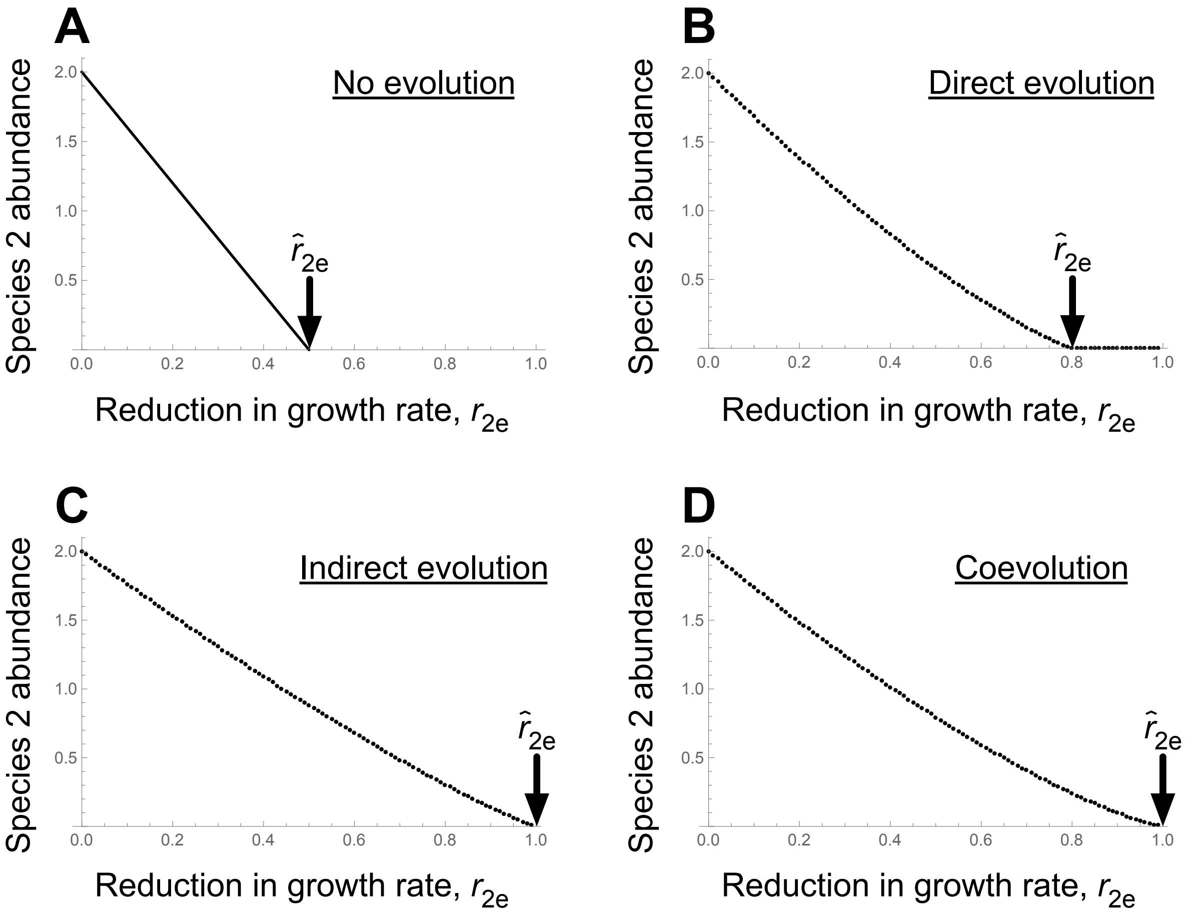

Figure S2. Bifurcation plots of indirect evolutionary rescue in competitive interactions. *X*-axis: Reduction in the basal intrinsic rate of increase of species 2 (r_{2e}). *Y*-axis: Species 2 abundance. (A) No evolution ($V_1 = 0$ and $V_2 = 0$). (B) Direct evolution ($V_1 = 0$ and $V_2 = 0.1$). (C) Indirect evolution ($V_1 = 0.1$ and $V_2 = 0$). (D) Coevolution ($V_1 = 0.1$ and $V_2 = 0.1$). Other parameter values are the same as in Fig. S1.

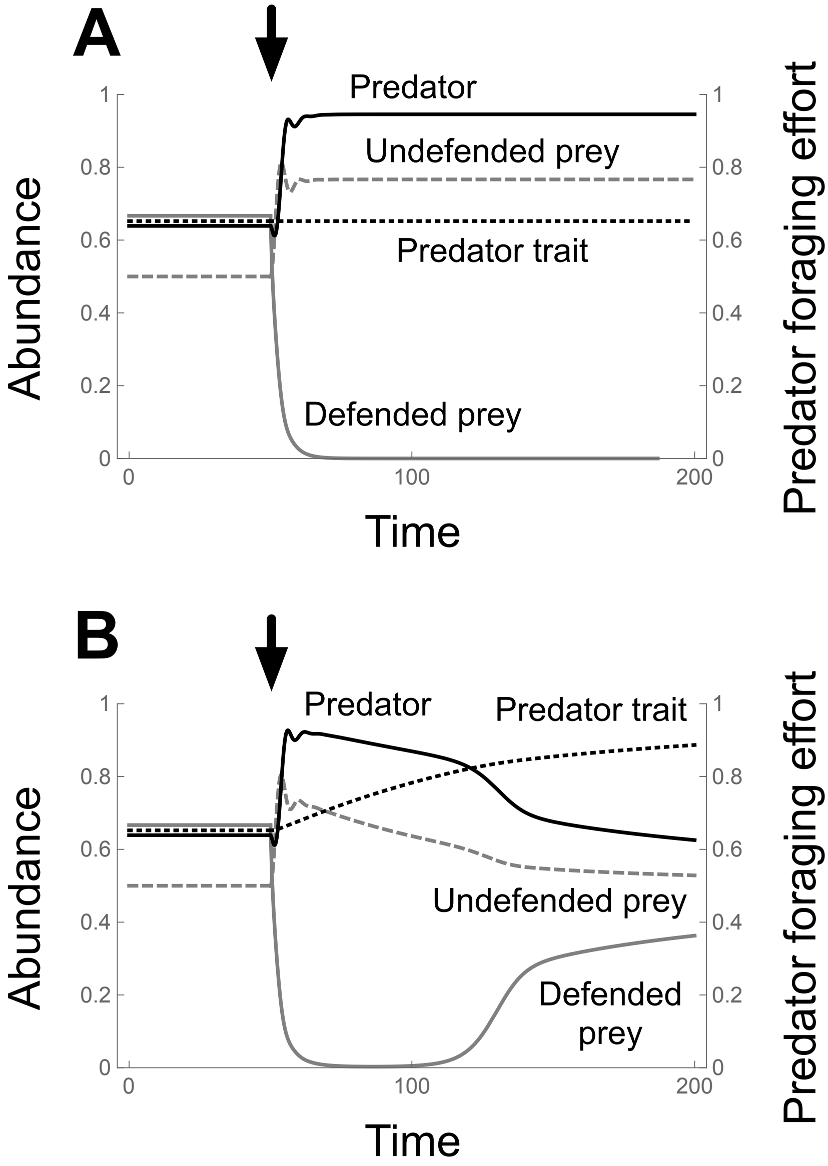

Figure S3. An example of indirect evolutionary rescue by adaptive foraging. The intrinsic rate of increase of defended prey (r_2) changed from 1.5 to 1 ($r_{2e} = 0.5$) at the timing of arrows. Gray solid lines: defended prey abundance, gray dashed lines: undefended prey abundance, black solid lines: predator abundance, black dotted lines: foraging effort to undefended prey. (A) No adaptation ($V_1 = 0$). (B) Adaptive foraging ($V_1 = 0.01$). Other parameter values are $b_1 = b_2 = d = 1$, $r_1 = G_1 = 2$, and $G_2 = 1.5$.

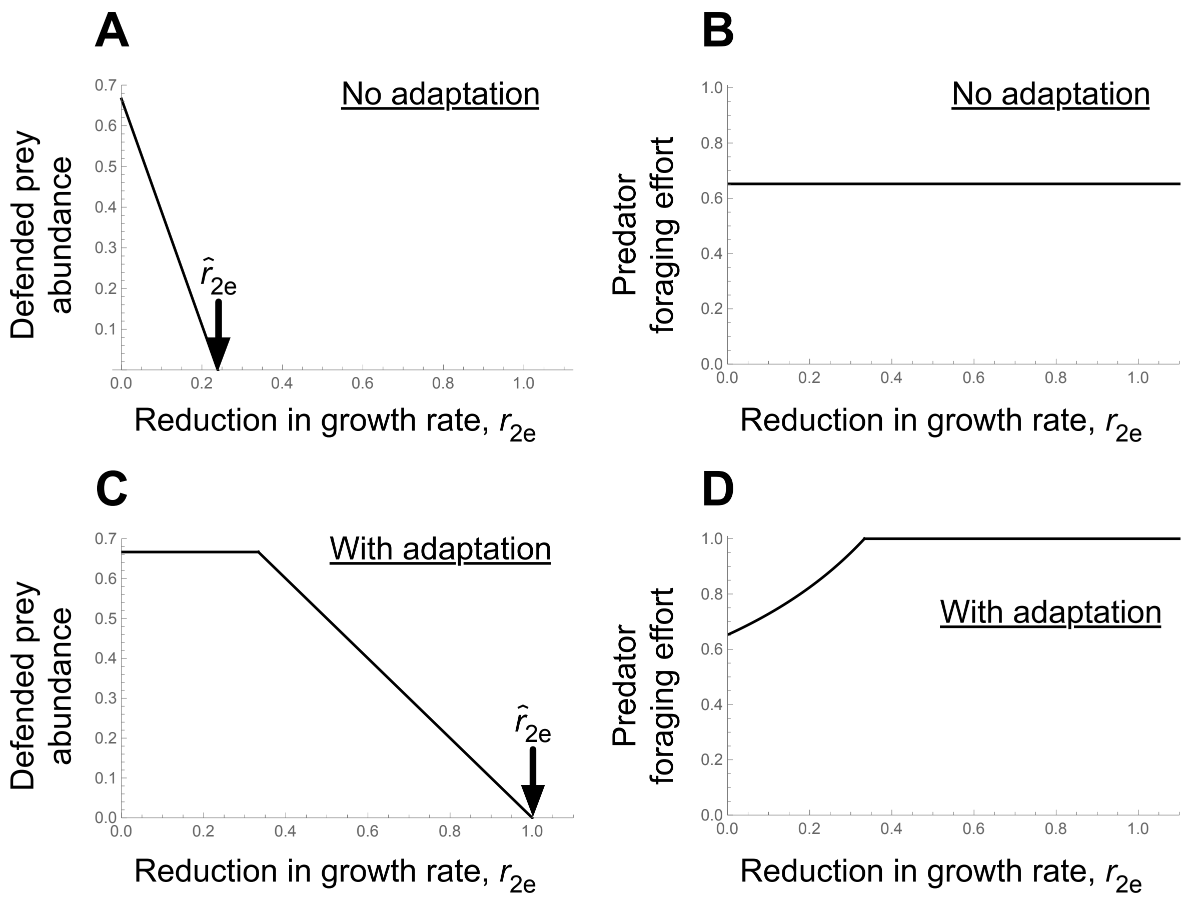

Figure S4. Bifurcation plots of indirect evolutionary rescue by adaptive foraging. *X*-axis: Reduction in the per capita growth rate of defended prey (r_{2e}). *Y*-axis: Defended prey abundance (A, C) or predator foraging effort on undefended prey (B, D). (A, B) Without adaptation, the defended prey goes extinct when $\hat{r}_{2e} = 0.24$. (C, D) With adaption in foraging effort, defended prey goes extinct when $\hat{r}_{2e} = 1.0$. Parameter values are the same as in Fig. S3.
